# Draft Genome Sequences of Two Acinetobacter baumannii Isolates from a Fatal Case of Necrotizing Fasciitis

**DOI:** 10.1128/MRA.00047-20

**Published:** 2020-03-26

**Authors:** Brock A. Arivett, Angella Charnot-Katsikas, Cindy Bethel, Steven E. Fiester, Luis A. Actis

**Affiliations:** a Department of Microbiology, Miami University, Oxford, Ohio, USA; b Department of Pathology, University of Chicago Medical Center, Chicago, Illinois, USA; c Department of Biomedical Sciences, University of South Carolina School of Medicine Greenville, Greenville, South Carolina, USA; d Department of Pathology, Prisma Health, Greenville, South Carolina, USA; University of Delaware

## Abstract

Carbapenem-resistant Acinetobacter baumannii is a bacterial pathogen with serious implications for human health and is recognized as an urgent threat by the Centers for Disease Control and Prevention (CDC). Total DNA from two A. baumannii clinical isolates collected over 3 days from a fatal case of necrotizing fasciitis has been sequenced to >30× coverage.

## ANNOUNCEMENT

Recently, the U.S. Centers for Disease Control and Prevention (CDC) stated that we are now in the postantibiotic era, due in part to the urgent threat of carbapenem-resistant Acinetobacter strains (1). The World Health Organization has also recognized that carbapenem-resistant Acinetobacter baumannii should be a critical focus for research and drug development (2). These statements place focus on this Gram-negative opportunistic nosocomial pathogen, which is often multidrug or pan-drug resistant and causes pneumonia, meningitis, soft tissue infections, and sepsis. (3). A. baumannii has rarely been isolated as the causative agent of necrotizing fasciitis (4); however, Charnot-Katsikas et al. described the isolation of A. baumannii, prior to antibiotic therapy, from two separate patients with necrotizing fasciitis. The A. baumannii strains isolated from both patients exhibited extensive drug resistance, including carbapenem resistance (4). The carbapenem resistance, unusual clinical appearance, and time of isolation of A. baumannii specimens such as these merit investigation. To that end, we investigated two isolates obtained from the male patient described in the report by Charnot-Katsikas et al. (4), one isolated during the course of infection, before antibiotic treatment, from a blood specimen (NFAb1) and the other isolated 3 days later, following antibiotic treatment, from postmortem tissue (NFAb2), in order to investigate the properties of drug-resistant A. baumannii leading to severe infection and death over the course of an infection.

Isolates were stored and genomes were prepared as described previously (5, 6). Briefly, total DNA was isolated, using the DNeasy blood and tissue kit (Qiagen, Valencia, CA, USA), from overnight Luria-Bertani broth cultures grown at 37°C with agitation. DNA quantity and quality were assessed using a NanoDrop 2000 spectrophotometer (Thermo Fisher Scientific, Wilmington, DE, USA). The SYBR Green (Life Technologies, Grand Island, NY, USA) standard curve method was used to estimate the DNA concentration for library preparation in a black 96-well plate (Corning, Tewksbury, MA, USA), and fluorescence values were obtained using a FilterMax F5 spectrophotometer with Multi-Mode Analysis software version 3.4.0.25 (Molecular Devices, Sunnyvale, CA, USA). DNA was simultaneously fragmented and adapter tagged using the Nextera XT kit (Illumina, Inc., San Diego, CA, USA), according to the manufacturer’s instructions. The DNA size distribution of the resultant libraries was determined using a Bioanalyzer 2100 high-sensitivity DNA analysis kit (Agilent Technologies, Santa Clara, CA, USA) with version B.02.08.SI648 software. Libraries were pooled and sequenced using the MiSeq 600-cycle kit version 3 (Illumina) to perform 300-bp paired-end sequencing on a MiSeq instrument (Illumina) according to the manufacturer’s instructions. All reads were trimmed by 15 bp from the 5′ end and filtered to remove reads with a quality score of less than Q20. *De novo* assembly was performed using Genomics Workbench version 7.5 with the Bacterial Genome Finishing module (CLC bio, Boston, MA, USA) on a workstation with an AMD Opteron 2.10-GHz 16-core processor and 128 GB of DDR3 ECC RAM. Genomes were annotated with the NCBI Prokaryotic Genome Annotation Pipeline (PGAP) version 4.10 using the best-placed reference protein set and GeneMarkS-2+ (7, 8).

For the A. baumannii NFAb1 isolate, the *de novo* assembly of 442,203 reads resulted in a 4,156,151-bp genome in 97 contigs (*N*_50_, 81,219 bp), with a GC content of 39.2%, containing 71 RNA genes and 4,032 genes, with 3,961 proposed coding sequences (CDSs). For A. baumannii NFAb2, the *de novo* assembly of 645,485 reads resulted in a 4,161,501-bp genome in 77 contigs (*N*_50_, 106,433 bp), with a GC content of 39.6%, containing 75 RNA genes and 4,027 genes, with 3,952 proposed CDSs.

### Data availability.

The first versions of the *de novo* whole-genome assemblies were deposited in GenBank under BioProject PRJNA261239, with accession number WKJR00000000 for A. baumannii NFAb1 and accession number WKJS00000000 for A. baumannii NFAb2. Sequence Read Archive accession numbers for the draft genomes are SRR10505817 and SRR10505816, respectively.
